# Comprehensive analysis of partial confounding clinical symptoms and treatment options of botulism: a case series

**DOI:** 10.3389/fneur.2025.1574807

**Published:** 2025-04-07

**Authors:** Xuanyu Huang, Zhiping Hu, Han Xiao, Yan Huang

**Affiliations:** ^1^Department of Neurology, The Second Xiangya Hospital of Central South University, Changsha, China; ^2^NHC Key Laboratory of Birth Defect for Research and Prevention (Hunan Provincial Maternal and Child Health Care Hospital), Changsha, China; ^3^Hunan Provincial Key Laboratory of Neurorestoration, Changsha, China

**Keywords:** botulism, atypical symptoms, neurophysiological studies, delay diagnosis, treatment

## Abstract

**Objective:**

To examine the clinical presentations, and therapeutic principles of botulism, with the goal of improving physicians’ understanding of the condition and refining treatment strategies.

**Method:**

A retrospective analysis was carried out on the clinical data of 8 patients with botulism, encompassing age, gender, etiology, delay diagnosis time, course of disease, clinical manifestations, auxiliary examinations, and treatment.

**Results:**

Among the 8 cases, 5 were female and 3 male, with the age ranging from 14 to 60 years. 5 cases were of iatrogenic poisoning, and 3 were of foodborne poisoning. Besides the classical clinical manifestations, some patients had atypical symptoms like intestinal obstruction, unilateral involvement, and consciousness disorder. Among the 8 cases, 6 had abnormal electrophysiological examination results. 6 patients had a delayed diagnosis of over 7 days, with disease course from 7 to 115 days, and 7 received treatment. All patients received symptomatic and supportive treatment. Moreover, 4 received invasive respiratory support, 3 received intravenous immunoglobulin injection, 1 received plasma exchange therapy, and 1 received antitoxin therapy. All patients were discharged with normal cranial nerve function, gastrointestinal function, muscle strength, and tone.

**Conclusion:**

Patients with botulism may exhibit atypical clinical symptoms, necessitating heightened vigilance from physicians. Neurophysiological studies are integral to the diagnostic process. Furthermore, symptomatic supportive treatment is essential for patients whose diagnosis has been delayed beyond 7 days. In conclusion, a comprehensive understanding of the clinical features, differential diagnostic criteria, and therapeutic options for botulism is essential for reducing disease duration, optimizing patient outcomes, and enhancing treatment efficacy.

## 1 Background

Botulism is a potent neurotoxin produced by *Clostridium botulinum* under anaerobic conditions, capable of blocking nerve signal transmission and resulting in muscle paralysis. There are seven known serotypes of botulinum toxin: A, B, C1, D, E, F, and G (1). The mechanism of action involves targeting the neuromuscular junction to inhibit the release of acetylcholine—a key neurotransmitter—thereby preventing muscle fiber contraction (2). In clinical practice, botulinum toxin is extensively utilized for the treatment of a variety of conditions, including chronic pain, muscle spasms, and cosmetic procedures (3). However, excessive administration of botulinum toxin can lead to toxicity, manifesting as symptoms such as acute symmetrical descending flaccid paralysis, cranial nerve palsy, and autonomic dysfunction in patients (4). When respiratory muscles are involved, it may progress to respiratory failure, posing a significant risk to life (5, 6). Consequently, timely diagnosis and management are essential for mitigating adverse outcomes. Currently, botulism is primarily diagnosed based on clinical manifestations and patient history. However, patients presenting with atypical symptoms can be challenging to identify in the early stages, leading to instances of misdiagnosis and delayed diagnosis (6, 7). Consequently, this study aims to conduct a retrospective analysis of the clinical features observed in 8 patients with botulism poisoning and to summarize both the clinical characteristics and treatment experiences associated with this disease. The ultimate goal is to enhance physicians’ understanding of botulism and optimize therapeutic strategies.

## 2 Methods

### 2.1 Research object

We conducted a retrospective study involving the clinical data of eight patients diagnosed with botulism who were treated at the Neurology Department of the Second Xiangya Hospital, Central South University, between January 2014 and October 2024. Due to the nature of this retrospective study, informed consent from patients and their families was waived. This study has received ethical approval from the Ethics Review Committee of the Second Xiangya Hospital, Central South University.

**Inclusion criteria:** (1) Adherence to the clinical diagnostic criteria for botulism (5). (2) Demonstration of gradual improvement following administration of antitoxin and other supportive treatments; if the condition continues to progress, alternative diagnoses should be considered. (3) Given that laboratory diagnosis of botulism is time-consuming, with complex diagnostic techniques and low sensitivity (8), it is primarily diagnosed based on clinical manifestations and medical history. Laboratory diagnosis serves only as an auxiliary criterion, where a negative result does not exclude the possibility of the diagnosis; this principle has also been applied in this study.

**Exclusion criteria:** (1) Neuromuscular junction disorders, including Myasthenia gravis and Lambert-Eaton syndrome, are excluded based on clinical features, neostigmine test, fatigue testing, repetitive nerve stimulation, and other relevant examinations. Central nervous system diseases such as acute cerebrovascular accidents, infection, and immune related encephalitis are ruled out through clinical assessment and cranial Magnetic Resonance Imaging (MRI). Additionally, paralysis resulting from peripheral neuropathy or myositis is excluded via clinical evaluation and neurophysiological studies. (2) Diseases resulting from other substance poisoning (e.g., carbon monoxide, organophosphorus compounds, etc.) are excluded based on contact history, clinical features, and laboratory examinations.

### 2.2 Research method

Comprehensive records were maintained for all patients, detailing their ages, genders, routes of poisoning, signs and symptoms, delay diagnosis time, course of disease, cerebrospinal fluid (CSF), neuroimaging, electrophysiological study, treatment and prognosis; these data were subsequently analyzed and summarized.

## 3 Results

### 3.1 General information

This study included a total of 8 patients with botulism, comprising 5 females (62.5%) and 3 males (37.5%). The age range of the patients was between 14 and 60 years. All patients had clear poisoning etiologies, with 5 cases (62.5%) attributable to iatrogenic factors and 3 cases (37.5%) to foodborne factors. All patients with iatrogenic botulism were female and developed symptoms following cosmetic injections of botulinum toxin. Furthermore, all foodborne botulism patients reported a history of consuming contaminated food such as home-prepared traditional processed stinky tofu, homemade honey, and dried beef (details in Table 1).

**Table 1:** Clinical characteristics of 8 patients in this study.

Patient	Age/sex	Delay diagnosis time (days)	Course of disease (days)	Etiology	Signs and symptoms											
					Pt	Emp	Pw	Pd	Fp	Dp	Lw	Rmi	Dy	Doc	Si	Gs
1	41/F	9	115	Iatrogenic	Y	N	N	N	Bilateral	Y	Y	Y	N	N	N	N
2	42/M	9	65	Foodborne	Y	Y	Y	N	N	N	N	Y	N	N	N	N
3	29/F	8	37	Iatrogenic	Y	Y	Y	N	Unilateral	Y	Y	Y	N	N	N	N
4	52/M	7	53	Foodborne	Y	N	Y	Y	N	Y	Y	Y	N	Y	N	N
5	37/F	4	28	Iatrogenic	Y	N	Y	N	Bilateral	Y	Y	N	N	N	N	N
6	44/F	15	22	Iatrogenic	N	N	Y	N	N	N	N	Y	N	N	N	N
7	60/F	4	7	Iatrogenic	Y	N	N	N	N	N	N	N	N	N	N	N
8	14/M	9	32	Foodborne	N	Y	Y	Y	Unilateral	Y	Y	N	N	N	N	Y

Doc, disturbance of consciousness; Dp, descending paralysis; Dy, Dysautonomia; Emp, extraocular muscle palsy; F, female; Fp, facial palsy; Gs, gastrointestinal symptoms; Lw, limb weakness; M, male; Pd, pupil dilation; Pw, palatal weakness; Pt, Ptosis; Rmi, respiratory muscle involvement; Si, sensory involvement.

### 3.2 Symptoms/signs and frequency of botulism patients

The primary symptoms and signs observed in the patients are presented in the following order: ptosis in 6 cases (75.0%), palatal weakness in 6 cases (75.0%), descending paralysis in 5 cases (62.5%), limb weakness in 5 cases (62.5%), respiratory muscle involvement in 5 cases (62.5%), facial nerve palsy in 4 cases (50.0%), extraocular muscle palsy in 3 cases (37.5%), pupil dilation (25.0%), disturbance of consciousness in 1 case (12.5%), and gastrointestinal symptoms in 1 case (12.5%). Among the four patients with facial nerve palsy, two exhibited unilateral involvement (details in Table 1).

### 3.3 Auxiliary examinations

All 8 patients underwent a comprehensive evaluation, including blood routine, CSF analysis, electrophysiological studies, and neuroimaging. Among these patients, 6 (75.0%) exhibited positive results on their blood routine, characterized by elevated white blood cell counts. All patients underwent lumbar puncture within 3 days of admission. Abnormal CSF findings were observed in 3 cases (37.5%). Among these, Case 1 exhibited elevated CSF protein levels. Notably, the CSF white blood cell count was elevated in all patients. Additionally, 6 cases (75.0%) had positive outcomes from neurophysiological testing. Specifically, cases 1, 2, and 7 displayed signs of neuromuscular junction impairment on electromyography (EMG), while cases 3, 4, and 8 showed incremental responses during repetitive nerve stimulation (RNS). Furthermore, all patients had negative results from cranial computerized tomography (CT) or MRI (details in Table 2).

**Table 2:** Clinical characteristics of 8 patients in this study.

Patient	Ancillary examination				Treatment
	Blood routine	CSF	Neuroimaging	Es	
1	Y	Y	N	Y	IVIG, PE, Mv, Symptomatic and supportive treatment
2	Y	Y	N	Y	Mv, Symptomatic and supportive treatment
3	Y	N	N	Y	Mv, Symptomatic and supportive treatment
4	Y	Y	N	Y	IVIG, Mv, Symptomatic and supportive treatment
5	Y	N	N	N	Antitoxin, Symptomatic and supportive treatment
6	N	N	N	R	Symptomatic and supportive treatment
7	N	N	N	Y	N
8	Y	N	N	Y	IVIG, Symptomatic and supportive treatment

CSF, cerebrospinal fluid; Es, electrophysiological studies; IVIG, intravenous immunoglobulin; Mv, mechanical ventilation; PE, Plasma exchange; R, refuse.

### 3.4 Course of disease and delay diagnosis time

This study included 8 patients, with a total disease duration ranging from 7 to 115 days. Cases 1, 2, 4, 6, and 8 were initially evaluated at external hospitals and diagnosed with conditions such as ‘vertigo,’ ‘Guillain-Barré syndrome,’ and ‘mechanical intestinal obstruction.’ Due to ambiguous diagnoses, exacerbation of clinical symptoms, or respiratory muscle involvement, these patients were subsequently transferred to the Second Xiangya Hospital of Central South University for further management. With the exception of cases 5 and 7, all other patients had experienced illness for a minimum of 7 days by the time they presented at our hospital (details in Table 1).

### 3.5 Treatment and prognosis

Among the 8 cases, with the exception of 1 patient (Case 7) who did not receive any treatment, the remaining 7 patients underwent various symptomatic interventions, including antibiotic therapy, neuroprotective nutritional support, gastric protection, hepatic support, and general nutritional assistance. Four patients (Cases 1, 2, 3, and 4) required mechanical ventilation due to respiratory muscle involvement. Additionally, three patients (Cases 1, 4, and 8) were treated with intravenous immunoglobulin therapy; Case 1 also received plasma exchange. Furthermore, one patient (Case 5) was administered antitoxin treatment. All patients were discharged with normal cranial nerve function, gastrointestinal function, muscle strength, and muscle tone (details in Table 2).

## 4 Discussion

In this study, we performed a retrospective analysis of 8 cases of botulism. Botulism is a rare yet potentially fatal condition (9), with approximately 200 cases reported annually in the United States (10), the etiological factors contributing to botulism primarily include: (1) foodborne; (2) wound-related; (3) iatrogenic; (4) inhalational; and (5) infant botulism as well as adult colonization botulism (5). Among the 8 cases analyzed, there were 5 female and 3 male patients. Excluding case 8, all other 7 patients were adults. From an etiological perspective, all five female patients experienced iatrogenic poisoning due to botulinum toxin injections, whereas the remaining three male patients suffered from foodborne poisoning caused by contaminated food such as home-prepared traditional processed stinky tofu, homemade honey, and dried beef. These foods are recognized as frequent causes of foodborne botulism in China (11). Overall, the etiological profiles of all patients in this study align with the typical etiologies documented in previous studies.

In accordance with existing literature (4, 12), the majority of patients (87.5%) in this study exhibited cranial nerve palsy as the initial symptom, with the predominant clinical manifestations being palatal weakness, ptosis, extraocular muscle palsy, and limb weakness. All patients presenting with limb weakness demonstrated a descending progression of the disease; specifically, it commenced with cranial nerve palsy and subsequently progressed to involve the limbs. Additionally, among the four patients who required mechanical ventilation due to significant respiratory muscle impairment, there was a notable association with an extended disease duration. This observation further corroborates previous findings that symptom severity is directly proportional to disease severity (13). All patients exhibited no sensory disturbances, which aligns with the anticipated outcome that botulinum does not impact sensory nerve fibers (14–16). However, case 8 presented with mechanical bowel obstruction as the initial symptom, characterized by vomiting, abdominal pain, and distension, thereby complicating early diagnosis significantly. Cases 3 and 8 demonstrated unilateral facial palsy, an atypical manifestation since symptoms of botulism are typically symmetrical; previous studies indicate that only 6–15% of patients exhibit asymmetry or unilateral neurological deficits (4, 17). We hypothesize that this may be attributable to differential effects of circulating toxins on both sides of the body due to factors such as anatomical asymmetry in cranial nerves or atherosclerosis. It is worth noting that in the absence of severe infection, hypoxia, and other underlying diseases, case 4 developed consciousness disorders, which may also be attributed to botulism, because the latest research indicate that botulinum toxin not only affects peripheral nerve terminals but may also exert indirect effects on the central nervous system through retrograde transport and neural plasticity (18, 19). Experimental research in rodent models has demonstrated that botulinum toxin can indeed cross the blood–brain barrier (20).

Botulism is typically diagnosed based on clinical symptoms and potential exposure history, followed by the detection of botulinum toxin in blood, stool, suspected food sources, or wound samples to confirm the diagnosis (10). Additionally, EMG can provide clinical evidence of neuromuscular transmission disorders prior to serological results, which holds significant diagnostic value (21). In patients with botulism, EMG may reveal nerve-muscle junction blockade, normal axonal conduction velocity, and reduced amplitude of compound motor action potentials (22). Approximately 60% of patients exhibit enhancement during RNS (23). Electrophysiological studies in cases 1, 2, and 7 revealed evidence of neuromuscular junction dysfunction, whereas RNS reports for cases 3, 4, and 8 indicated progressive findings that facilitated both diagnosis and differential diagnosis from other neuromuscular disorders such as Guillain-Barré syndrome and Myasthenia gravis. In contrast, routine laboratory tests for patients—including complete blood count, CSF, and neuroimaging—were generally unremarkable. Case 1 exhibited a separation of cerebrospinal fluid protein and cells, attributed to the patient’s concurrent Guillain-Barré syndrome. In the other cases, abnormal findings included elevated white blood cell counts in both blood and CSF, which may be associated with pulmonary infections and concurrent meningitis. Pulmonary infection is a common complication of botulism that can exacerbate pre-existing respiratory difficulties, leading to rapid deterioration of the patient’s condition (24). Therefore, we propose that active prevention and treatment of infections are essential through enhanced assessment of swallowing function, improved turning and coughing assistance, and dynamic monitoring of temperature, respiratory sounds, sputum volume, and color.

The nonspecific nature of symptoms, along with potential omissions and inaccuracies in patient-reported histories and the complexities associated with differential diagnosis, often results in misdiagnosis, oversight, or delays in the diagnosis of botulism. In a retrospective analysis of 332 suspected cases of botulinum toxin poisoning from 1980 to 2016, alternative diagnoses were considered in 274 instances, with Guillain-Barré syndrome (*n* = 99) and Myasthenia gravis (*n* = 76) being the most prevalent. Additionally, these cases may be misdiagnosed as various common and rare conditions, including cerebrovascular accidents, Lambert-Eaton syndrome, meningitis, encephalitis, and tick paralysis (4). In this study, 5 of the 8 patients experienced misdiagnosis or delays in diagnosis. For instance, case 1 initially presented with extraocular muscle palsy and ptosis, which rapidly progressed to involve facial and cervical muscles within a span of 24 h. Subsequently, the patient exhibited symptoms of limb weakness and respiratory muscle involvement. CSF analysis revealed protein-cell dissociation. Serum antibody testing for peripheral nerve disorders indicated the presence of IgG antibodies against GM2, GM3, and GT1a. Consequently, upon admission, we diagnosed this patient with Guillain-Barré syndrome and initiated treatment with plasma exchange and immunoglobulin therapy. Due to ineffective early treatment, we only discovered that the patient had concealed a history of botulinum toxin injection before the onset of the disease after receiving electrophysiological study results and repeatedly inquiring about the medical history. Therefore, based on the comprehensive medical history and examination results, we diagnosed the patient with concurrent botulism. The clinical presentation of this patient presented a considerable diagnostic challenge, as, in addition to the classic subtype, Guillain-Barré syndrome also manifests in a variant characterized as pharyngeal-cervical-brachial type mimicking botulism. This variant may occur alongside anti-GT1a antibodies (although detection is not widespread) and typically presents with descending paralysis rather than the classic ascending pattern observed in typical GBS (25, 26). Consequently, misdiagnosis, missed diagnosis, or delayed recognition of botulism can impose an additional economic burden on the patient, delay treatment initiation, and adversely affect prognosis. We propose that improvements in diagnostic accuracy can be achieved through the following measures: 1. Collecting medical history as comprehensively as possible to minimize omissions and errors; 2. Familiarizing healthcare professionals with the characteristic signs and symptoms of botulism while giving careful consideration to differential diagnoses.

Antitoxin represents the most critical treatment for botulism poisoning, as it can bind to toxins that have not yet attached to synaptic receptors in the bloodstream, thereby facilitating their removal from circulation (27, 28). Numerous studies have demonstrated that early administration of antitoxin therapy can mitigate respiratory muscle involvement, shorten disease duration, and reduce mortality risk (28–31). However, other research indicates that while antitoxin may halt disease progression, it cannot reverse the effects of poisoning once established (32). The potential benefits of antitoxin therapy are minimal for patients whose symptoms have persisted for more than 7 days since very little toxin is detectable in the body after this period (5). Furthermore, if only supportive care (including mechanical ventilation) is provided, nearly all patients can survive even without antitoxin intervention (30). In this study, out of the 8 patients included, except for case 7 who did not receive treatment due to a milder condition, the remaining 7 patients received respiratory support, antibiotics, nutritional support for the nerves, and other symptomatic supportive treatments. In addition, cases 1, 4, and 8 received plasma exchange and immunoglobulin therapy due to previous misdiagnosis. Only Case 5 received antitoxin treatment, as all patients except for Case 5 and Case 7 had been ill for more than 7 days and some had respiratory muscle involvement when they came to our hospital for treatment. Considering that antitoxin has a certain incidence of adverse reactions (28), we only provided symptomatic supportive treatment. However, all patients recovered and were discharged, with a definite therapeutic effect. Therefore, the indications and timing for the use of antitoxin certainly deserve further investigation by researchers.

Given that antitoxin cannot reverse paralysis (5) and may also lead to adverse effects such as bradycardia, tachycardia, and cardiac arrest (28), researchers have explored alternative methods for treating botulism. While some patients with botulism have undergone plasma exchange therapy, there was no significant effect (33). Additionally, cholinergic agonists (such as guanidine and 3,4-diaminopuridine) have been investigated for their potential in treating botulism due to their established use in other neuromuscular disorders; however, their efficacy remains limited (34–36). Although polyethylene glycol preparations have been proposed to enhance the excretion of toxins from the gastrointestinal tract, there is currently no evidence supporting their benefit (37). A meta-analysis has indicated that thus far, no specific treatment method other than botulinum antitoxin has demonstrated effectiveness (30). Consequently, future research in this field may focus on the development of novel therapeutic agents, including additional cholinergic agonists.

Our study has several limitations. First, the sample size of this study is small, and most indicators show no statistically significant differences, which limits the ability to draw conclusions. Therefore, further validation is warranted through larger sample sizes and multi-factor analyses in future research. Second, due to the limited sample size, we combined iatrogenic and dietary botulinum toxin poisoning for the analysis of symptoms/signs and treatment outcomes. Although both share a similar pathogenesis, their etiologies differ, presenting certain limitations. Finally, this study only conducted a retrospective statistical analysis of the in-hospital information from previous botulism cases and lacked follow-up data.

## 5 Conclusion

This study provides a comprehensive summary of the clinical features and treatment outcomes of botulism patients treated at our institution over the past decade. Certain patients present with atypical symptoms, such as initial bowel obstruction, unilateral involvement, and altered consciousness, necessitating heightened vigilance from physicians. In addition to pathogen detection, electrophysiological studies play a crucial role in diagnosis. For patients experiencing diagnostic delays exceeding 7 days, symptomatic supportive therapy alone can yield favorable outcomes, further underscoring its significance and highlighting the need for further investigation into the indications and timing of antitoxin administration. In conclusion, a comprehensive understanding of the clinical features, differential diagnostic criteria, and therapeutic options for botulism is essential for reducing disease duration, optimizing patient outcomes, and enhancing treatment efficacy. This study contributes to deepening physicians’ comprehension of the condition, thereby increasing diagnostic accuracy and optimizing treatment strategies. Future research in this domain may concentrate on the pathophysiological mechanisms, the neutralization mechanisms of antitoxins, and development of novel therapeutic agents for botulism. These areas require further investigation to facilitate deeper exploration.

## Data Availability

The original contributions presented in the study are included in the article/supplementary material, further inquiries can be directed to the corresponding authors.
